# Developing the Senses Framework to support relationship-centred care for people with advanced dementia until the end of life in care homes

**DOI:** 10.1177/1471301216682880

**Published:** 2016-12-06

**Authors:** Julie Watson

**Affiliations:** School of Health in Social Sciences (Nursing Studies), Edinburgh Centre for Research on the Experience of Dementia, University of Edinburgh, Edinburgh, UK

**Keywords:** advanced dementia, care homes, relationship-centred care, embodied selfhood, social death

## Abstract

People with advanced dementia living in care homes can experience social death before their physical death. Social death occurs when a person is no longer recognised as being an active agent within their relationships. A shift is required in how we perceive people with advanced dementia so that the ways they continue to be active in their relationships are noticed. Paying attention to embodied and interembodied selfhood broadens the scope and opportunities for relationships with people with advanced dementia, acting as a counter to social death. This has the potential to improve the quality of care, including end of life care, of people with advanced dementia in care homes. This study examined the role of embodied and interembodied selfhood within care-giving/care-receiving relationships in a specialist dementia care home. Empirical findings and their implications for the development of relationship-centred care and the Senses Framework in care homes are discussed.

## Introduction

People with advanced dementia often struggle to maintain relationships and can experience isolation and, therefore, social death before their physical death (Alzheimer’s Society, 2013). Social death is the cessation of the individual as a social actor, or an active agent in other people’s lives (Brannelly, 2011; Froggatt, 2001; Glaser & Strauss, 1965; Sudnow, 1967). Lyman (1993) suggests that assumptions about the effect of dementia on personhood/selfhood directly affect the way people with dementia are perceived by others, the quality of their relationships, the quality of their care and the quality of their life (Lyman, 1993). A common assumption is that, due to cognitive impairment, advanced dementia gradually takes away the person. Advanced dementia has been described as the ‘unbecoming’ of self (Fontana & Smith, 1989) and the ‘very splintering of the sedimented layers of being until ultimately there is nothing left’ (Davis, 2004, p.375). Adopting this view will mean social death is more likely to happen for people with advanced dementia.

As people with dementia approach the final phase of their life, Brannelly (2011, p.665) suggests that, ‘being treated as socially dead robs the self of the dignity of a meaningful and bearable death’. It can mean that people with dementia become viewed as passive recipients, or objects, of care. As people with dementia face the final phase of their life, which can be a protracted period of dependency over many months or even years (Murray, Kendall, Boyd, & Sheikh, 2005; van der Steen et al., 2013), challenging social death is an important consideration in their care. To achieve this, a shift is required in how we perceive people with advanced dementia so that the ways they continue to be active in their relationships are noticed. Such a shift is suggested by Kontos (2004) who draws attention to the bodyliness of human beings and that we are in the world through the vehicle of our body. In an ethnographic study of 13 residents with moderate to advanced Alzheimer’s disease in a long-term care facility, she observed that movements of the body, facial expressions (smiles and frowns), eye behaviour (blinking, winking, direction and length of gaze and pupil dilation) and posture, all carried implication and meaning: ‘they played a large role in inter-personal communication ….and were intentional, communicative, informative and interactive’ (Kontos, 2003, p.835).

Kontos (2004) suggests that these observations of selfhood persisting in the face of severe cognitive impairment is because selfhood resides in corporeality, or in the whole body, not just the brain; the body ‘knows’ how to perform pre-reflectively and does not rely on cognition (Kontos, 2005). Recognising embodied selfhood in dementia care means that behaviours that may have previously been overlooked become noticed, increasing the scope and opportunities for interpersonal relationships and improved quality of care (Kontos & Martin, 2013).

The philosophy of Merleau-Ponty shows how the body remains a constituent of selfhood until death. He says that: ‘… to be a ‘self’ is to be a living creature that is in the world through the vehicle of one’s body’ (Merleau-Ponty cited in Matthews, 2002, p.69). The body is, at one and the same time, a phenomenal (sentient or feeling) body and an objective (sensible or feelable) body. Merleau-Ponty refers to this as a body-subject. The body remains a constituent of selfhood, a body-subject, until death and this is an important consideration in dementia care.

Merleau-Ponty (1968) says that the human body as a visible thing is contained within the full spectacle of visible things in the world, including other people, and describes an intertwining of one with the other. Methodologically, this is important for understanding the experience of people with dementia who are unable to talk. It challenges the assumption that the mind is an inner world separate from the body: it is possible, at least in part, to understand another’s experience because it is visible. Hughes quotes the philosopher Wittgenstein saying: ‘the human body is the best picture of the human soul’ (Hughes, 2011, p.50).

This intertwining with each other has important implications for interactions in dementia care and a practical outworking of this is given by Zeiler (2013). Using the example of singing together, Zeiler (2013) shows how Gladys, who has dementia, can express herself through singing with Naomi (https://www.youtube.com/watch?v=CrZXz10FcVM). Zeiler (2013) suggests we hold each other in personhood through our inter-embodied capabilities. In this example, this depends on both Gladys and Naomi having an openness to each other, perceiving each other’s bodily expressions and responding, creating a shared space of dynamic engagement where something of each person ‘springs forth’ in the joint activity which would not be seen without it (Zeiler, 2013, p.138).

In the UK, one-third of people with dementia live in care homes (Alzheimer’s Society, 2013) and 55% of people with dementia die in care homes (Murtagh et al., 2012). In care homes, positive relationships are understood to be central to good care (Davies & Brown-Wilson, 2007; Goodman, Amador, Elmore, Machen, & Mathie, 2013; Robinson, Reid, & Cooke, 2010). Nolan, Davies, Brown, Keady, and Nolan (2004) and Nolan, Brown, Davies, Nolan, and Keady (2006) advocate relationship-centred care which shifts the focus from just being on the person receiving care to creating an enriched environment which supports relationships and where the needs of the staff and family are also considered. Nolan et al. (2006) developed and empirically tested the Senses Framework; they suggest that the person receiving care, family carers and paid carers should all experience relationships that promote the six Senses (Box 1). The fundamental premise is that good care which is relationship-centred can only be delivered when the Senses are experienced by all parties (Ryan, Nolan, Reid, & Enderby, 2008).

**Box 1.** Outline of the Senses Framework (Nolan et al. 2006 with permission).

**Sense of security – To feel safe**:
For older people: Attention to essential physiological and psychological needs, to feel safe and free from threat, harm, pain and discomfort. To receive competent and sensitive care.
For staff: To feel free from physical threat, rebuke, or censure. To have secure conditions of employment. To have the emotional demands of work recognised and to work in a supportive but challenging culture.
For family carers: To feel confident in knowledge and ability to provide good care without detriment to personal well-being. To have adequate support networks and timely help when required. To be able to relinquish care when appropriate.
**Sense of belonging – To feel part of things:**
For older people: Opportunities to maintain and/or form meaningful and reciprocal relationships, to feel part of a community or group as desired.
For staff: To feel part of a team with a recognised and valued contribution, to belong to a peer group, a community of gerontological practitioners.
For family carers: To be able to maintain/improve valued relationships, to be able to confide in trusted individuals to feel that you are not in this alone.
**Sense of continuity – To experience links and connection:**
For older people: Recognition and value of personal biography; skilful use of knowledge of the past to help contextualise present and future. Seamless, consistent care delivered within an established relationship with known people.
For staff: Positive experience of work with older people from an early stage of career, exposure to good role models and environments of care. Expectations and standards of care communicated clearly and consistently.
For family carers: To maintain shared pleasures/pursuits with the care recipient. To be able to provide competent standards of care, whether delivered by self or others, to maintain involvement in care across care environments as desired/appropriate.
**Sense of purpose – To have a personally valuable goal(s) to aspire to:**
For older people: Opportunities to engage in purposeful activity facilitating the constructive passage of time, to be able to identify and pursue goals and challenges, to exercise discretionary choice.
For staff: To have a sense of therapeutic direction, a clear set of goals to which to aspire.
For family carers: To maintain the dignity and integrity, well-being and personhood of the care recipient, to pursue (re)constructive/reciprocal care.
**Senses of achievement – To make progress towards these goals:**
For older people: Opportunities to meet meaningful and valued goals, to feel satisfied with ones efforts, to make a recognised and valued contribution, to make progress towards therapeutic goals as appropriate.
For staff: To be able to provide good care, to feel satisfied with ones efforts, to contribute towards therapeutic goals as appropriate, to use skills and abilities to the full.
For family carers: To feel that you have provided the best possible care, to know you’ve ‘done your best’, to meet challenges successfully, to develop new skills and abilities.
**Sense of significance – To feel that ‘you’ matter as a person:**
For older people: To feel recognised and valued as a person of worth, that one’s actions and existence are of importance, that ‘you matter’.
For staff: To feel that gerontological practice is valued and important, that your work and efforts ‘matter’.
For family carers: To feel that one’s caring efforts are valued and appreciated, to experience an enhanced sense of self.

While the Senses Framework does recognise the importance of each person in a relationship experiencing a sense of significance and feeling that they matter as a person, it does not explicitly engage with the effects of dementia on personhood, the implications of this for relationships, and the potential for social death, as described above.

Hughes (2013) argues that we could dismiss the philosophy of personhood/selfhood as too theoretical if it weren’t for commonly heard comments such as ‘he’s not the person I married’ and the persistent reports of poor quality dementia care in some hospitals and care homes in relation to meeting even basic needs such as hydration and nutrition or being taken to the toilet (Cohen, 2011, 2015; Francis, 2013).

The purpose of this paper is to examine, through the lenses of embodied and interembodied selfhood, the ways care staff and people with dementia living in a care home relate to each other and how these theories can develop the Senses Framework and promote high quality relationship-centred care with people with dementia until the end-of-life.

## Research questions

This paper, drawn from a PhD study (Watson, 2015), addresses the questions:

- How is embodied and inter-embodied selfhood enacted in day-to-day care in a care home?
- What is the role of embodied and inter-embodied selfhood in ensuring people with advanced dementia remain active agents in their relationships until the end of life?
- What role does embodied and inter-embodied selfhood play in promoting relationship-centred care?

## Ethics

Ethical approval was granted by the NHS Research Ethics Committee (Scotland A) (Reference no. 12/SS/0116).

## The setting

The research took place in ‘Primrose Hill’ (a pseudonym): a care home for 40 people with dementia. It volunteered to participate on hearing about the study through a research network. It met the purposive sampling criteria for the study in terms of size, focus on dementia and quality of care on last inspection (graded 4 (good) or above on all quality themes).^1^ It was a social care home, therefore not registered to employ nurses or provide nursing care. Nursing care was provided by community nurses and medical care by General Practitioners (GPs) visiting the care home. Eight staff were on duty during the day and four during the night. The staff were at various stages of attaining Scottish Vocational Qualifications in Health and Social Care (Scottish Social Services Council http://www.sssc.uk.com/) and undergoing dementia care training through the Promoting Excellence Framework. In Scotland, this outlines the knowledge and skills all health and social care staff should aspire to achieve in supporting people with dementia and their families (Scottish Government, 2010).

## Methodological approach

I adopted an ethnographic approach with an appreciative intent. I aimed to understand what worked in interactions between care staff and residents in day-to-day life in the care home. My aim was not to ignore problems but rather to gain a deep appreciation of what practice entails and to understand what works within the context of care homes, while retaining a critical eye. This sits alongside a body of work on dementia care and care homes which focus on problems and barriers to good practice, which are well documented (e.g. Thune-Boyle et al., 2010).

Ethnography is where the researcher immerses themselves in a social situation and collects real world observations in a pragmatic, reflexive and emergent way, producing insights about actions and events placed in context (Greenhalgh & Swinglehurst, 2011). To participate in the life of what is being studied, the role of one of the actors in the field is taken up and explored (Jenkins, 1994). I adopted the role, at least partially, of a care assistant, helping to care for residents, both in terms of social interaction in communal areas such as sitting rooms, and also assisting with direct hands-on care in bedrooms^2^ with a selection of residents as described below.

My inter-embodied interactions with residents centred around care were revealing and helped me not only to get to know them, but also to appreciate what it was like to relate to them, what was difficult, and, what worked. I judged what worked by the way residents responded, particularly their expressions, either verbal or non-verbal, of whether they seemed settled or distressed. This was based on the notion that understanding another’s experience does not depend solely on them talking about it, but rather it is visible, in their facial expressions, body posture and behaviour. My own active involvement in caring was a way of getting *inside* the caring relationship, giving me some insight into the way selfhood is revealed in embodied and inter-embodied ways, how care staff learn how to relate to residents, and a basis from which to explore the staff’s experience in more depth.

Tedlock (2000) describes this as a shift from participant observation to the observation of participation. This is a shift from an objectifying methodology to an intersubjective methodology where the ‘researcher and ‘the researched’ appear in the ethnographic narrative. It means that research can be understood not only in terms of ‘what’ has come to be known but also ‘how’ it has come to be known (Altheide & Johnson, 1998; Etherington, 2007), making visible the position of the researcher and demystifying the construction of knowledge (Pillow, 2003).

## Recruitment and consent

All care home residents were eligible for inclusion for general participant observation. Focussed participant observation was aimed at residents who were more likely to have six months or less to live based on the following criteria: difficulties with mobility, in need of help with all activities of daily living, incontinence, no consistently meaningful conversation, poor food intake, recent weight loss (Gold Standards Framework Prognostic Indicator Guidance, 2016). End of life prognostic indicators for dementia are poorly defined and staff were also asked to consider which residents they felt were declining.

All residents in the care home lacked capacity to give written informed consent; therefore, it was legally required from a guardian or welfare attorney with power to consent to the adult’s participation in research or, where there was no such guardian or welfare attorney, from the adult’s nearest relative, in accordance with the Adults with Incapacity (Scotland) Act, 2000.

Forty invitation letters were sent by the care home manager to residents’ guardian, welfare attorney or relative. Initially the number of people invited to take part in focussed observation was limited to a subset of four as I was uncertain how families might feel about their relative being involved in this way. All four relatives gave consent. One of these residents died within three months. To increase opportunities for focussed observation, a further four relatives were invited to give consent for their relative to take part and all four relatives gave consent. It is good practice to check, if at all possible, if the person with dementia themselves would like to take part and what the basis for making the decision is for that person in terms of how they usually communicate day to day. The basis for ongoing process consent throughout the study was established and followed for each resident for whom written consent was given (Dewing, 2002).

All staff were eligible for inclusion in all aspects of the study and were given written information sheets and opportunities to discuss the study.

## Study participants

Written informed consent was given by relatives for 20 residents, 17 female and 3 male, for general observation which included 8 for focussed observation. Their mean age was 90 years old. Length of stay in the care home ranged from one year to nine years with an average of three years.

Of the 47 staff, 33 consented to participate, 28 day staff and 5 night staff. All 33 took part in observation while a subset of the 33 took part in interviews and/or group discussion as time allowed within the busyness of the care home. Twenty-five were female and eight were male. Specific roles were known to myself but not used in presented data to protect anonymity. The full spectrum of roles was represented in those who participated: managers, team leaders, care workers, care assistants and ancillary staff.

Information on those who did not take part was not collected so it is not possible to say how staff or residents who took part differed from those who did not take part, except to say that some staff who declined to take part said they were too busy.

## Methods

From November 2012 to August 2013, I visited the care home twice weekly. There were 68 visits, plus 10 during the recruitment phase, with a total of 207 hours spent in the care home around the clock. Each visit lasted on average 3 hours and entailed time spent doing general and focussed participant observation as opportunities arose. While there were some opportunities to ask staff questions while working with them, these were limited by concerns about talking over residents. I therefore, also prearranged time with staff to do interviews and group discussions during my visits.

- Observation (total – 195 hours): I undertook two levels of participant observation: firstly, general participant observation in communal areas of daily life and interactions between consenting staff and residents, while at the same time I was interacting socially with residents, e.g. chatting or taking part in activities such as church services or coffee mornings; secondly, focussed participant observation where I worked with staff as they did hands-on care, such as washing and assisting with meals, with consenting staff and residents. In the first two months, as I got to know residents and staff, I spent the majority of time on 3-hour visits doing general participant observation. In the remaining eight months, I spent approximately half of each 3 hour visit on focussed participant observation. A small amount of time was also spent on non-participant observation such as looking at residents’ case notes. I took short notes during fieldwork and later developed these into detailed descriptive fieldnotes interspersed with reflexive notes, reflections, issues to follow up, emerging themes and references to other literature.
- Twenty-four interviews (total – 10.5 hours) with care staff (3 pairs and 18 one-to-one) lasting between 15 minutes and 45 minutes. Interviews were digitally recorded with permission and transcribed verbatim. Issues arising from the participant observation were explored, guided by the questions in Appendix 1.
- Three group discussions (total – 2 hours) with care staff (2 groups of 4 and 1 group of 5) focussing on the care of residents dying in the home during the time of the study. Each group discussion was structured around a reflective cycle (St Christopher’s Hospice, 2013) and I used questions to guide the discussion (Appendix 2). The discussion was digitally recorded with permission and transcribed verbatim.
- Examination of care plans and daily records kept on residents participating in focussed observation. Particular attention was paid to aspects of care foregrounded in daily notes and whether embodied aspects of personhood were written about, for example the way residents communicated in embodied ways. It also helped me keep track of what was happening on days I was not in the care home, for example if someone had been unwell. Notes were written up more fully as fieldnotes and formed part of the overall analysis.

## Confidentiality

All fieldnotes and transcripts were anonymised with pseudonyms and identifying information such as specific roles removed. All data were securely stored in locked filing cabinets and password protected computers in the university. However, it is difficult to guarantee confidentiality in ethnographic research and those connected with the care home may recognise people and situations within the findings.

## Analysis and interpretation

Fieldnotes, interview and group discussion transcripts were managed with NVIVO qualitative data analysis software. A coding framework was developed to examine themes. Chronological case studies of all 20 participating residents were created, compiling all the data held in relation to them. This helped form a more coherent ‘story’ of each person over time as a way of revealing something new. It gave residents a louder ‘voice’ within the data, bringing them to the fore as key players in the caring relationship, distilling the key factors shaping their relationships with the care staff.

Mattingly (1998) and Mattingly and Garro (2000) in ethnographic research with occupational therapists describe emergent narratives as the stories that are not told but enacted or embodied, the ‘something’ that people hope will happen during therapy that is healing. The data were examined for enacted or embodied emergent narratives and what the ‘something’ might be that both residents and staff hoped might happen in their encounters.

The next step was to redescribe what was happening in the care home in the language of social scientific discourse (Blaikie, 2010). This merges interpretative validity, what the data is interpreted to mean for those involved, with theoretical validity by going *beyond* what is valid for individuals while remaining grounded in data to support any claims (Whitaker, 2010). This meant examining the data again using theory on embodied and inter-embodied selfhood as analytical lenses (Jenkins, 2013; Kontos, 2003, 2004; Zeiler, 2013).

## Trustworthiness of the findings

Plausibility is achieved by transparency in analysis and interpretation and the presentation of multiple voices and perspectives. An analysis diary was used to track the analysis and interpretative insights. This, and the sharing of emerging findings with participating staff as a means of member checking, adds to the interpretative validity of the findings. The integration of theory on the body into the design, fieldwork and analysis of the study provides theoretical validity.

## Findings^3^

Three overarching themes which shaped the face-to-face relationship between care staff and residents in the care home emerged:

- Hands-on care or ‘body work’, which includes care in the dying phase but also during the prolonged period of care before this phase is reached.
- Recognising and supporting selfhood.
- Witnessing and responding to distress.

## Body work

During the fieldwork period, 1 resident could get up and wash and dress independently, 14 could wash and dress with prompting, and 25 required almost full assistance with up to six people requiring hoisting. The predominant focus of the handover, which all staff got when they came on duty, was on bodily functions, particularly bowel and bladder function. This was to identify subtle changes which may have indicated, for example, a urine infection which could lead to confusion, which as well as being upsetting for the person, could be very upsetting for everyone else. Quickly identifying brewing infections was important, not only for the well-being of the person affected, but for maintaining a calm atmosphere and social order in the care home. Consequently, a considerable part of the staff’s day was taken up with body work (Twigg, 2000; Twigg, Wolkowitz, Cohen, & Nettleton, 2011): washing, dressing, feeding, monitoring and documenting bodily functions, taking residents to the toilet, or moving residents around the building. Body work was one of the main points of contact between staff and residents and, therefore, a key component of their relationship.

Taking an appreciative stance, and moving beyond first impressions which may have indicated a routinized, task-orientated approach, to a closer examination of body work, and the way people with dementia were positioned in these interactions, was revealing. Many care staff saw body work as an opportunity to connect with the person and foster a sense of belonging:

I still make a point of it's not just a task at hand, it's, this is time where you are interacting one-to-one and it's good for them … even if they are not really talking, it's still that one-to-one time that they are getting … (Edith, Staff Member, Interview, 11/3/13)

Paying attention to what residents were communicating in embodied ways was an important element of body work:

… it is hard to explain how you can read that there is pain in (Penny’s) face, you know, but the fact that she was rubbing and she didn’t look as happy as usual, then she’s not … we … we got the doctor to see her and she actually has quite, you know, bad knees and I try to say, they will, you know, be swollen and things, so she’s on like regular pain killers now. (Zoe, Staff Member, Interview, 7/3/2013)

Body work was not always straight forward:

… they might not feel comfortable with you giving them a shower, or anyone giving them a shower … but, like, you’ve got to give them a shower, you know, you’ve got to help them in that way. (John, Staff Member, Interview, 19/4/13)

Such tensions were often most apparent at mealtimes, when residents, who often could not verbalise their wishes, would communicate by spitting food out or turning their faces away, as I experienced while assisting Maureen:

(Maureen) ate her fruit quite happily. She then got porridge with cream and sugar. She spat out the first spoonful onto the tablecloth. She was saying no and she was not smiling but had a closed stern look on her face and was looking away and not making eye contact. (Fieldnote – Maureen p.36 15/11/12)

Maureen normally enjoyed porridge. Scenarios such as this could cause dilemmas for care staff:

… you don’t force somebody if they don’t want to eat and they are making that clear to you whether it’s pushing your hands away or spitting food out you’ve got to respect that, so that’s tough from our point of view because you want them to get their nutrition, you want them to have fluids but at the same time you have to respect their wishes … (Hilary, Staff Member, Group Discussion, 18/1/13)

Dilemmas around food and drink were intensified when a resident was recognised as becoming less well but there remained some uncertainty around whether they were dying, or might get better. Weight loss and reduced oral intake in someone who has advanced disease and is frail are a natural part of the dying process (Gold Standards Framework, 2016). The fieldnote below shows care staff struggling to encourage Annie, a 97-year-old who died less than three weeks later, to drink and take her medication:

Three people described as ‘failing’ at handover – one of them was Annie. She was in the dining room for over two hours (this morning at breakfast). Throughout this time Edith (Staff Member), spent a lot of time with her trying to encourage her to open her mouth and swallow a high-calorie drink and her medication. Annie seemed very reluctant to take any fluids into her mouth or swallow. Annie barely had her eyes open and looked worn out. (Fieldnote p.185 26/4/13)

These scenarios show that hands-on body work is not simple (Twigg, 2000). People with advanced dementia are not passively receiving care, but actively involved in the care-giving/care-receiving interaction: they are not objects but body-subjects.

## Recognising and supporting selfhood

Care staff had to learn about the selfhood of residents in two interrelated ways. Firstly, they had to learn about their care needs and how to practically care for them, such as how to get them up in the morning and what kind of approach was required to enable this. ‘Experiencing’ residents led to embodied knowing:

I’ve done courses and things but I’ve learned through doing and experiencing them, like if you were to read all the information about them and then come in … it’s not the same, although the information is right … (Vera, Staff Member, 26/6/13)

This ‘phenomenology’ of caring is rooted in ordinary lived experience and active involvement in a two-way interaction between the staff and the residents. Secondly, the staff had to learn things about the person, such as knowing about their life and what kind of person they were, helping to create a sense of shared identity. Important within this was a recognition that the person in front of them had not always been like this.

I think that knowing a little bit about what they did makes you just … I don’t know, you just don’t look at them as just, okay, that’s one of the residents, you look at them, that’s Penny, you know, that’s what she did (talks about all the things Penny did) … so it helps you to, you know, to identify, to just care for … I started to realise that there is more than just … just the Penny that I know … there is much more. (Zoe, Staff Member, Interview, 7/3/13)

Knowing things about a person’s life were also described as ‘*handles to hold onto*’ which helped them connect person-to-person, and to care. The embodied knowing was combined with knowledge of the person’s culture in ways which helped caring run more smoothly. For example, Hilda was a lady who could walk but would get stuck on one spot. Anne’s advice below to me was to hold her as if doing a Scottish dance:

Anne suggested I walk with my right arm holding Hilda’s right arm like doing the Gay Gordon’ as this seems to help her to keep going forwards and go in the right direction. (Fieldnote p.198 3/5/13)

Anne’s advice was effective and created a ‘shared space of dynamic intercorporeal engagement’ (Zeiler, 2013, p.136) with Hilda to achieve forward movement, drawing on Hilda’s ‘sedimented’ embodied memories of Scottish dancing. This level of connection and sense of continuity with the past may be more difficult for staff from a different cultural background, in this example, unfamiliar with Scottish dancing.

During ‘body work’ it was often the small details about a person, aspects of biographical continuity and embodied selfhood, which made the difference to the smoothness of an interaction:

… if Annie doesn’t have her socks, like, if you take her stockings off and you’ve not got her (bed)socks ready to put on then she’ll …… kick off. (Kathy, Staff Member, Joint Interview, 21/3/2013)

… those silly wee things should be in the care plan, like, I like to wear bed socks. (Nita, Staff Member, Joint Interview, 21/3/2013)

Lawton (2000), in a study of selfhood in the context of advanced cancer, suggests that bodily deterioration leading to dependency and the loss of agency is implicated in the process which leads to social death. It seemed that staff could fall into the habit of seeing residents as passive rather than active agents, leading to over-caring and the unnecessary withdrawal of bodily capability from a person (Hakanson & Ohlen, 2014):

I asked Sandra (a resident) if she would like a wet cloth for her hands which she took from Anne (a staff member) and washed her hands with. I then gave Sandra a dry one. Initially she seemed bemused but then did dry her hands with it. Anne was surprised as she seemed to think Sandra wouldn’t know what to do with it. Anne said ‘it’s amazing how old habits remain’. (Fieldnote p.202 9/5/2013)

When staff tried to do things for residents which they could still do themselves, failing to recognise embodied abilities which are part of selfhood, residents could become upset, indicating that it may have been undermining their selfhood:

(The carer) couldn’t get Hilda to stand up. John (Staff Member) went to help her. Hilda was resisting and saying no. They were trying to encourage her to stand by taking her arms under the shoulders. Eventually John just asked her to stand up and she did it and said something like ‘I can do it’. (Fieldnote p.237 5/6/2013)

However, at other times, help was welcomed and did not cause distress, which suggests that in itself, getting help does not undermine selfhood:

Betty’s mouth was dirty from her lunch. I went and got her a cloth and gave it to her saying it was to wipe her mouth. She wiped it a little bit but then gave the cloth back to me. I asked if she wanted me to wipe her mouth and she nodded and let me do it. (Fieldnote p.204 14/5/2013)

In the later stages of dementia when the person was very dependent, often the presence of the person with dementia would slowly emerge when the staff were open to the person, paying attention and expecting a response:

‘I think it’s really hard, I mean Penny, that’s heart breaking isn’t it because I’d love for her to be able to talk to me and tell me all the cool stuff that she used to do, it’s hard. But it’s really rewarding, like the other night I put her to bed and said ‘goodnight Penny, God Bless’ and she said ‘good night’ back and I was like ‘oh we’ve just had a conversation’ and that was really really cool ….that made me smile lots ….(Debra, Staff Member, Interview, 9/5/13)

This encounter fosters a sense of achievement for Debra and a sense of significance and belonging for both Debra and Penny. Each one is attending to each other’s signs of attention which is a ‘pre-reflective process of social attunement’ (Zeiler, 2013, p.138); Penny ‘knows’ how to interact despite losing many of her cognitive abilities. These subtle connections between the carer and the cared for are rewarding for both and as Jenkins (2013) suggests can help dissolve the distinctions between the carer and the cared-for, creating a ‘we’. The staff recognised this, ‘feeling’ the response in an embodied way, even when a resident was dying, referred to by staff as ‘palliative’:

Hilary: You almost feel it … you maybe sense a change in the person’s breathing, they come more relaxed or calm or the eyes open, they look directly at you, close again, just wee things. (Staff Member, 29/1/2013)

However, on another occasion, a woman with dementia who was slowly dying was described by a staff member as a ‘shell’, indicating an understanding of the self as being gone, and overlooking the body as a constituent of the self. While relationships inevitably change as dementia progresses and death approaches, it is important to recognise that an embodied reciprocal interchange persists until the end of life. Attending to these subtle interactions and embodied aspects of selfhood is crucial to challenging social death and alleviating the distress this can cause in advanced dementia.

## Witnessing and responding to distress

Many care staff were attuned to the physiological causes of distress such as pain, constipation or delirium and sought to alleviate these as soon as possible. Yet, during observations there was evidence of persistent visible distress, such as unsettledness, with people getting up and down out of their chairs, or calling out for help and seeking one-to-one attention:

I was chatting away and she was light hearted and everything and I says ‘come on up you get lazy bones’ and stuff like that and she just laughs and she stands up, what have you, … and then the second I went into her bathroom to clean her teeth she started groaning and I said ‘Sandra, why are you making this noise, it’s a terrible noise, what’s all this about Sandra? …’you went away and left … I didn’t want you to go away and leave me’… ‘but Sandra I have to, I’m only in the bathroom cleaning your teeth but there’s other people that need my attention as well … I will come back and see you’. (Gemma, Staff Member, Interview, 3/6/13)

Sandra above seems to lack a sense of security. Consistent relationships, where residents got to know the ‘face’ of the staff, helped create a sense of security and continuity:

… She couldn't remember my name or anything but she always knew she knew my face, she didn't know how, um, and I always seemed to brighten her day. So if I came in and she was in an awful mood and she had been shouting at everybody, I'd come in and she'd go, oh Bunty, and she always used to call me Bunty …. because with dementia obviously it's hard because it's hard to get through to people to try and reassure them and help them. (Edith, Staff Member, Interview, 11/3/13)

‘She knew my face’ points to the importance of the inter-embodied nature of consistent relationships, not relying on cognition, such as remembering names.

Sometimes the source of distress seemed beyond what staff could do anything about and the embodied experience of being in a busy care home contributed to the agitation of some residents:

I think it’s too many people, too big a place and I think that gets her a bit more agitated I think … I think she just needs … even when you try and give her something else to do it doesn’t last for very long … I don’t know, we have tried so many things … (Vera, Staff Member, Interview, 26/6/13)

This type of distress impacted on the care-giving/care-receiving relationship. It jeopardised the sense of purpose and achievement of the staff and left some staff feeling horrible:

But the horrible thing about this job is you can’t sit all day with one resident, like we have forty people, so it’s harder that way. (John, Staff Member, Interview, 19/4/13)

In care home life, this is an important consideration as the staff recognised an interdependency between their feelings and the feelings of the residents:

… and they feel as well, if you are nervous they know that … I walked in one day, I was just walking in and Ingrid said to me ‘oh, is there anything wrong?’ and I thought no, I didn’t say anything and what was it, I can’t even remember what it was, something quite simple but she actually picked up so much and then I think that’s quite odd so ….they are very perceptive and they feel your feelings, you know, again they are very experienced people … (Brenda, Staff Member, Interview, 29/1/13)

This mutual exchange of feelings between bodies, or intercorporeally, has an effect on both the residents and staff, and it could be positive:

(If) I’m feeling rubbish or whatever … I like to go and sit beside Penny (resident) and … you cuddle in or whatever. That gives something for each of you, she … I think she likes it too because sometimes she puts her hand on yours or she’ll play with your hair or … and it’s quite nice, I feel quite calm sitting beside her … (Vera, Staff Member, Interview, 26/6/13)

There is an ‘interplay of intersubjectivities’ (Twigg et al., 2011, p.174); care staff simultaneously affect a resident’s experience and are affected by it (Farber & Farber, 2006). When this exchange of feelings is negative, it has important implications for the caring relationship. Fifteen residents died during the time of the study and the awareness of death as a source of distress for residents was recognised by staff:

I felt at the end Joanna was actually … I think she knew she was dying and she was frightened … to be alone. (Muriel, Staff Member, Interview, 11/3/13)

Muriel described caring for Joanna, who was frightened to be alone, as ‘draining on the team’. It was difficult for the staff, at times, to know what to do with the strong emotions raised by caring for people with advanced dementia with phrases such as ‘*(controlling my emotion) costs me sometimes a lot’*, and ‘*you might have nightmares*’ indicating the high level of emotional work required by staff in care homes. There were also signs of emotional detachment with comments such as ‘*I seem to be getting very detached to it*’, ‘*I’m just burned out with it*’.

## Discussion

The frailty of residents alongside the presence of dementia had significant implications for the way that care staff interacted with residents in Primrose Hill. It meant their relationship largely revolved around body work such as washing, dressing, taking to the toilet, and assisting with food. Hands-on care such as washing and feeding has since the 1990s in the UK become part of social care, as opposed to health care, and is generally considered to be ‘unskilled’ or less important work. Nurses, or those wishing to advance their career in social care, have become increasingly distanced from this type of work (Twigg, 2000).

Putting an analytical focus on body work revealed that body work is not a simple thing but is complex, often raising tensions around so-called simple tasks such as washing and dressing or assisting with food. In my own participation in body work, such as assisting at mealtimes or helping people get up in the morning, I sensed the pressure to ensure people ate well or did not lie in bed. This was considered to be quality care. However, I also came to see how overlooking, or overriding, embodied expressions of autonomy, such as spitting food out, contributed to social death because it did not recognise that people with advanced cognitive impairment continue to have embodied autonomy.

In the context of advanced dementia, considering the response to care and the autonomy of the person with dementia must pay more attention to the place of embodied autonomy if care which challenges social death and promotes comfort is to happen. Dekkers (2010) suggests that bodily autonomy, or the wisdom of the body, may be used as a guide when difficult moral and ethical decisions, including end-of-life decisions, are to be made with and for those who are deemed to have lost the capacity to make decisions. Bodily actions such as pushing away a spoon, spitting out medication, alongside facial expressions or bodily defensive movements should be taken seriously as communicative, interactive, informative and intentional (Kontos, 2003) when treatment decisions are to be made. This comes with the caution that it is a complex area of care which requires expertise, a relational context, teamwork and open and honest communication with families to avoid abuse.

There is no doubt, as Bailey, Scales, Lloyd, Schneider, and Jones (2015) found, that the severity of impairments of people with advanced dementia provide a constant challenge to care staff in recognising the persistence of selfhood. This makes the caring relationship in care homes complicated. However, as I observed daily life in the care home, I often saw authentic human encounters between care staff and residents which were visibly enriching for both, and experienced these myself in my participation in the life of the care home. There were many examples where care staff did not experience themselves as caring for a mere body, or an object, but ‘experienced’ people with dementia as experiencing body-subjects. In this study, even those who were classified as ‘palliative’, which was a euphemism for ‘dying’, were still seen as an experiencing human being by the majority of the staff, even in the last few days of life. This was because people with dementia retained a basic openness to others which enabled them to engage in a way that carers ‘felt’ with their own bodies.

The majority of care staff recognised the responsiveness of the residents; that they inhabit the world, and experience it emotionally, rather than being ‘objects’ in the world (Matthews, 2002). Merleau-Ponty (1962, p.186) argued that it is ‘through my body that I understand other people, just as it is through my body that I perceive things’. These insights from the care staff and the way that through ethnographic approaches the visible experiences of people with dementia can be articulated are very important because so often people with advanced dementia are assumed to be unaware of their surroundings or what is happening.

Recognising and supporting the continuity of the selfhood of residents played an important part in body work in that the past life of the resident was important to their present situation. Knowing about residents helped care staff to know how to care for them. Empirically, in this study, embodied aspects of selfhood, such as wearing certain items of clothing, described as appearance work by Twigg and Buse (2013), were found to be important. As Kelly (2010) suggests, these embodied aspects of selfhood need more attention within dementia care.

In this study, I was interested in how the notion of inter-embodied selfhood ‘springing forth in joint activity’ (Zeiler, 2013) was evident in the care-giving/care-receiving relationship as a way of seeing the person with dementia’s remaining capabilities and supporting selfhood. There were indeed examples of this happening. When a resident who rarely speaks is spoken to, has their hand held and is included and as a result they reciprocate with a word of thanks or a smile, something of that person’s remaining embodied selfhood and their agency ‘springs forth in joint activity’. They are being treated as an experiencing body-subject rather than a body object. When a carer assists someone to get washed by enabling them to do the things they can still do, such as washing their face or combing their hair, abilities sedimented within their body at a pre-reflective level through repeated motor activity over years (Merleau-Ponty cited in Zeiler, 2013), something of that person, their remaining embodied abilities, ‘springs forth in joint activity’ in a way that it would not if the carer did everything for them. This helped avoid confrontations during hands-on body work and may also, through supporting selfhood, bolster self-esteem. It was also important for some residents, rather than struggling to be independent, to have their need for help acknowledged, recognising that needing care is not a weakness but part of being human (Leece & Peace, 2010; Tronto, 1993).

When carers have the right mind set they can attend to the often subtle signs and bodily expressions indicating the person they are caring for is present. This then leads them to be open to connecting with them and attending to their response to receiving care. The relationship is asymmetrical in that it depends on the care staff intentionally taking the initiative. Care staff in care homes need encouragement to be more confident that they are connecting with the real person whose selfhood, in their experience, is persisting, despite cultural norms which would suggest otherwise for those with advanced cognitive impairment. Crucially, this would mean that people with dementia are given an ‘agential role (in) the care assemblage’ (Jenkins, 2013, p.9) which helps challenge social death. At the crux of this is the importance of enabling people to come together to create the conditions in which inter-embodied selfhood can ‘spring forth in joint activity’.

Many aspects of body work cannot wait, for example if someone needs the toilet, or is feeling ill and needs help (Cohen, 2011). The care staff in Primrose Hill had no choice but to prioritise body work. While care of the body is care of the self and can bring comfort both physically and emotionally, the busyness of a care home with 40 dependent residents with dementia and other co-morbidities means that care staff have little time with residents beyond hands-on body work. This at times led to distressing situations for both residents and staff.

This study shows an interconnection between the feelings of care home staff and residents. If people with dementia are able to ‘feel the feelings’ of those caring for them, emotional detachment by carers as a way of coping with unrelieved distress will be felt by residents, increasing their sense of isolation and contributing to their social deaths, and their distress (Bailey et al., 2015). This is counterproductive in the caring relationship and undermines the humanity of both care givers and care receivers; both are fully experiencing body-subjects, not body objects passively receiving care, nor machines passively delivering care. Hughes, Louw and Sabat (2006) suggest that care staff retain their humanity by being supported to remain engaged with, and respecting, those who rely on them for their care.

It is beyond the scope of this paper but it is important to acknowledge that face-to-face relationships in care homes are shaped by the wider socio-political context. Cohen (2015: no page) suggests that care staff have become the ‘residual casualties unable to compensate for the structural problems’ endemic in care homes, in a society which does not take seriously the central place of care in human life. More attention needs to be paid to staffing levels and training needs that take full account of the complexity of care for people with dementia in care homes.

While it is understandable for carers to adopt avoidance behaviour, it is unacceptable and counterproductive for carers to be unsupported (Lawrence, Samsi, Murray, Harari, & Banerjee, 2011; Weiner, 2006). Bailey et al. (2015) highlight the importance of organisations taking responsibility for the emotional lives of staff. This underlines the importance of the Senses Framework as a practical guide for individual staff, and organisations, to produce the conditions and the culture in care homes in which relationship-centred care can occur, for the benefit of staff and residents. The findings above are now used to incorporate the philosophical concepts of embodied selfhood and inter-embodiment into the Senses Framework for care homes for people with dementia (Box 2).

**Box 2.** Implications for practice – Development of the Senses Framework.

**Sense of security – To feel safe:**
*For people with dementia:*
At individual staff and organisational level – Attending to the embodied ways that people respond to their environment and their care. Recognising that people perceive their environment in an embodied way and that the busyness of a care home may cause them to feel insecure or frightened.
At individual staff level – Recognising the embodied ways that people with dementia communicate physical pain and other symptoms which can be alleviated, even if they can’t communicate verbally.
*For staff:*
At organisational level – Recognising that the feelings of people with dementia and those who care for them are intertwined. This means staff need organised emotional care providing opportunities to talk as a team and critically reflect on emotionally or ethically challenging situations and decisions, including around end of life care.
**Sense of belonging – To feel part of things:**
*For people with dementia and staff:*
At individual staff and organisational level – Taking responsibility for creating space for authentic human encounters and the person to emerge, both the person with dementia and the care giver. Recognising that the ‘space’ can be during hands-on body work or social situations.
*For people with dementia:*
At individual staff level – Being open to connecting, recognising the person with dementia is open to connecting, recognising the person with dementia as fully present and continuing to be able to take part in relationships in a meaningful way.
**Sense of continuity – To experience links and connection:**
*For people with dementia:*
At individual staff level – Recognising the importance of gathering a detailed biography of the person to include their usual routines and embodied identities, e.g. wearing bedsocks.
At organisational level – Recognising the importance of knowing ‘the face’ of another person, even if names are forgotten, and considering this in daily work allocation.
*For people with dementia and staff:*
At individual staff and organisational level – Establishing a shared sense of identity which helps to connect person to person, including supporting staff from other countries who may have a different cultural background to connect.
**Sense of purpose – To have a personally valuable goal(s) to aspire to:**
*For staff:*
At individual staff and organisational level – Recognising that body work, supporting selfhood and witnessing and responding to distress are key facets of an overall therapeutic goal of dementia care in care homes and are valuable.
At organisational level – Affirming that authentic human encounters in all aspects of care, including social activities and body work, matter.
*For people with dementia:*
At organisational level – Reframing from focussing on independence to focussing on interdependence and supporting remaining embodied abilities, while recognising that dependency is not a failure but part of being human.
At individual staff and organisational level – Recognising and supporting embodied autonomy of the person with dementia and drawing on this in decision making day-to-day and at the end of life.
**Sense of achievement – To make progress towards these goals:**
*For staff:*
At organisational level – Recognising that hands-on body work is not a task to be achieved for the benefit of the organisation but a key aspect of supporting selfhood and caring for the whole person.
At individual staff level – Articulating and documenting examples of embodied and inter-embodied selfhood to enhance their legitimacy and ensure they are recognised by all those involved in the process of care: families, service managers, commissioners, regulators and policy makers, as well as point of care staff.
*For staff and people with dementia:*
At individual staff and organisational level – Attending to the subtle signs which can easily go unrecognised, indicating that the person with dementia is present and connecting in a meaningful way as a means of providing comfort, even in the last days of life. Affirming that this is an authentic human encounter that matters and that in itself is an achievement for the care giver and care receiver.
**Sense of significance – To feel that ‘you’ matter as a person:**
*For people with dementia*:
At individual staff level – Attending to the person with dementia’s response and taking this seriously, recognising them as fully present.
*For people with dementia and staff:*
At individual staff and organisational level – Recognising the significance of the body and using embodiment as a resource.
At individual staff and organisational level – Recognising the intertwining of the feelings of residents and staff and that both matter in the caring relationship.

## Study limitations

This research was carried out in one care home and cannot claim to be generalizable. It does, however, provide a contextualised understanding of the caring relationship between care staff and people living and dying with dementia in a care home, providing a basis to compare other studies and a framework for thinking about how to develop relationship-centred care for people with dementia in care homes. The focus of this study was on the relationship between care staff and residents; inclusion of families and examination of relationships between staff was beyond its scope. However, it is acknowledged that all three types of relationships are critical in relationship-centred care and in practice require balanced attention (Koloroutis, 2004; Tresolini, 1994).

## Conclusion

Using the ‘body’ as an analytical lens, this study shows the ways that people with advanced dementia continue to be fully present experiencing people, interacting with those who care for them, until the end of life. It also shows what we can learn from care home staff through their experience of the human encounter with people with advanced dementia. The findings presented challenge the dominant societal view that people with advanced dementia are socially dead and provides a deeper understanding of the complex nature of dementia care in care homes. Developing the Senses Framework provides practical guidance for supporting relationship-centred care until the end of life for people with advanced dementia.

## Notes

1 The Care Inspectorate regulates and inspects care services in Scotland. They inspect the quality of care and support, environment, staffing, management and leadership. Each of these quality themes is assessed on a scale of one to six, where one is unsatisfactory and six is excellent (Care Inspectorate, 2016).

2 I am also a registered nurse and worked in a hospice for 11 years with people with cancer.

3 All names are pseudonyms.

4 Who benefits from these changes is in italics. Who needs to make these changes is underlined.

## Appendix 1. Interview outline^4^

- Could you tell me about a relationship you have with a resident in the home which you think is good?
- What makes it good?
- What do you think it might mean to the resident?
- What does it mean to you?
- What do you think helps it go well?
- What do you do, or what is your role in making a good relationship with residents?
- What do residents do that contributes to good relationships?
- What role do families play in helping you form relationships with residents?
- Is connecting with residents while you are assisting them different from connecting with them at other times? In what way?
- How do you continue to relate well to residents as they become less well?
- How do you continue to relate well to residents as they are dying?
- What difference does it make to you as a care worker to relate well or have a good connection to residents?

## Appendix 2. Group discussion guide

For each death:

Tell me what happened –

- Did they go downhill suddenly or at what point did you realise they were dying? Who realised? What was the decision making process – how did you decide what the plan of care would be?
- What time did they die – at night?
- What were they like when they were dying? Settled? Any medication needed?
- What involvement if any was there from the GP/DN?
- How were the family supported?
- What about other residents – do they get told?
- What about after the death – how do you deal with the body, undertakers etc.?
- Funeral? Memorial?
- What do you think you did well?
- Anything you would like to have done differently?
- What was challenging?

## References

[bibr1-1471301216682880] Adults with Incapacity (Scotland) Act. (2000). Retrieved from www.legislation.gov.uk/asp/2000/4/contents.

[bibr2-1471301216682880] Altheide D. Johnson J. (1998) Criteria for assessing interpretive validity in qualitative research. In: Denzein N. Lincoln Y. (eds) Collecting and interpreting qualitative materials, Thousand Oaks, CA: Sage, pp. 283–312.

[bibr3-1471301216682880] Alzheimer’s Society. (2013). Low expectations report: Attitudes on choice, care and community for people with dementia in care homes. Retrieved from www.alzheimers.org.uk/site/scripts/download_info.php?fileID=1628.

[bibr4-1471301216682880] Bailey S. Scales K. Lloyd J. Schneider J. Jones R. (2015) The emotional labour of health-care assistants in inpatient dementia care. Ageing and Society 35: 246–269.

[bibr5-1471301216682880] Blaikie N. (2010) Designing social research, 2nd ed. UK: Polity Press.

[bibr6-1471301216682880] Brannelly T. (2011) Sustaining citizenship: People with dementia and the phenomenon of social death. Nursing Ethics 18(5): 662–671. 21893577 10.1177/0969733011408049

[bibr7-1471301216682880] Care Inspectorate. (2016). Retrieved from www.careinspectorate.com/index.php/about-us.

[bibr8-1471301216682880] Cohen R. L. (2011) Time, space and touch at work: Body work and labour process (re)organisation. Sociology of Health and Illness 33(2): 189–205. 21299568 10.1111/j.1467-9566.2010.01306.x

[bibr9-1471301216682880] Cohen, R. L. (2015). Crises in care – Compassion and body work. *Discover Society*, January 2015 Issue 16. Retrieved from http://discoversociety.org/2015/01/03/crises-in-care-compassion-and-body-work/.

[bibr10-1471301216682880] Davis D. H. J. (2004) Dementia: Sociological and philosophical constructions. Social Science and Medicine 58: 369–378. 14604622 10.1016/s0277-9536(03)00202-8

[bibr11-1471301216682880] Davies, S., & Brown-Wilson, C. (2007). Creating a sense of community. In *National Care Homes Research & Development Forum*. *‘My Home Life’ Quality of Life in Care Homes: a review of the literature*. London: Help the Aged.

[bibr12-1471301216682880] Dekkers W. (2010) Persons with severe dementia and the notion of bodily autonomy. In: Hughes J. C. Lloyd-Williams M. Sachs G. A. (eds) Supportive care for the person with dementia, Oxford: Oxford University Press, pp. 253–262.

[bibr13-1471301216682880] Dewing J. (2002) From ritual to relationship: A person-centred approach to consent in qualitative research with older people who have a dementia. Dementia 1(2): 157–171.

[bibr14-1471301216682880] Etherington K. (2007) Ethical research in reflexive relationships. Qualitative Inquiry 13(5): 599–616.

[bibr15-1471301216682880] Farber A. Farber S. (2006) The respectful death model: Difficult conversations at the end of life. In: Katz R. S. Johnson T. G. (eds) When professionals weep, London: Routledge, pp. 221–236.

[bibr16-1471301216682880] Fontana A. Smith R. W. (1989) Alzheimer’s Disease victims: The ‘unbecoming’ of self and the normalisation of competence. Sociological Perspectives 32: 35–46.

[bibr17-1471301216682880] Francis, R. (2013). Independent inquiry into care provided by Mid Staffordshire NHS Foundation Trust January 2005 – March 2009. Retrieved from www.gov.uk/government/publications/independent-inquiry-into-care-provided-by-mid-staffordshire-nhs-foundation-trust-january-2001-to-march-2009.

[bibr18-1471301216682880] Froggatt K. (2001) Life and death in English nursing homes: Sequestration or transition? Ageing and Society 21(3): 319–332. 10.7748/nop.13.9.8.s9 27702331

[bibr19-1471301216682880] Glaser B. G. Strauss A. L. (1965) Awareness of dying, New York, NY: Aldine.

[bibr20-1471301216682880] Gold Standards Framework Prognostic Indicator Guidance. (2016). Retrieved from www.goldstandardsframework.org.uk/cd-content/uploads/files/General%20Files/Prognostic%20Indicator%20Guidance%20October%202011.pdf.

[bibr21-1471301216682880] Goodman C. Amador S. Elmore N. Machen I. Mathie E. (2013) Preferences and priorities for ongoing and end-of-life care: A qualitative study of older people with dementia resident in care homes. International Journal of Nursing Studies 50(12): 1639–1647. 23866093 10.1016/j.ijnurstu.2013.06.008

[bibr22-1471301216682880] Greenhalgh, T., & Swinglehurst, D. (2011). Studying technology use as a social practice: The untapped potential of ethnography. *BMC Medicine, 9*(45), 1–7. 10.1186/1741-7015-9-45 PMC3108909 21521535

[bibr23-1471301216682880] Hakanson C. Ohlen J. (2014) Meanings and experiential outcomes of bodily care in a specialist palliative context. Palliative and Supportive Care 13(3): 625–633. 24762673 10.1017/S147895151400025X

[bibr24-1471301216682880] Hughes, J. C., Louw, S. J., & Sabat, S. R. (Eds.). (2006). *Dementia: Mind, meaning and the person*. Oxford: Oxford University Press.

[bibr25-1471301216682880] Hughes J. C. (2011) Thinking through dementia, Oxford: Oxford University Press.

[bibr26-1471301216682880] Hughes J. C. (2013) ‘Y’ feel me?’ How do we understand the person with dementia? Dementia 12(3): 348–358. 24336857 10.1177/1471301213479597

[bibr27-1471301216682880] Jenkins T. (1994) Fieldwork and the perception of everyday life. Man 29(2): 433–455.

[bibr28-1471301216682880] Jenkins N. (2013) Dementia and the inter-embodied self. Social Theory and Health 12: 125–137.

[bibr29-1471301216682880] Kelly F. (2010) Recognising and supporting self in dementia: A new way to facilitate a person-centred approach to dementia care. Ageing and Society 30(1): 103–124.

[bibr30-1471301216682880] Koloroutis M. (2004) Relationship-based care: A model for transforming practice, USA: Creative Healthcare Management.

[bibr31-1471301216682880] Kontos, P. C. (2003), *Embodied selfhood: An ethnography of Alzheimer’s disease*. Unpublished PhD Thesis, University of Toronto, Canada.

[bibr32-1471301216682880] Kontos P. C. (2004) Ethnographic reflections on selfhood, embodiment and Alzheimer’s disease. Ageing and Society 24: 829–849.

[bibr33-1471301216682880] Kontos P. C. (2005) Embodied selfhood in Alzheimer’s disease: Rethinking person-centred care. Dementia 4(4): 553–570.

[bibr34-1471301216682880] Kontos P. C. Martin W. (2013) Embodiment and dementia: Exploring critical narratives of selfhood, surveillance and dementia care. Dementia 12(3): 288–302. 24336852 10.1177/1471301213479787

[bibr35-1471301216682880] Lawrence V. Samsi K. Murray J. Harari D. Banerjee S. (2011) Dying well with dementia: Qualitative examination of end of life care. The British Journal of Psychiatry 199: 417–422. 21947653 10.1192/bjp.bp.111.093989

[bibr36-1471301216682880] Lawton J. (2000) The dying process: Patients’ experience of palliative care, London and New York: Routledge.

[bibr37-1471301216682880] Leece J. Peace S. (2010) Developing new understandings of independence and autonomy in the personalised relationship. British Journal of Social Work 40: 1847–1865.

[bibr38-1471301216682880] Lyman K. E. (1993) Day in, day out with Alzheimer’s: Stress in caregiving relationships, Philadelphia: Temple University Press.

[bibr39-1471301216682880] Mattingly C. (1998) Healing dramas and clinical plots: The narrative structure of experience, Cambridge: Cambridge University Press.

[bibr40-1471301216682880] Mattingly, C., & Garro, L. C. (2000). *Narrative and cultural construction of illness and healing*. Berkeley and Los Angeles: University of California Press.

[bibr41-1471301216682880] Matthews E. (2002) The philosophy of Merleau-Ponty, UK: Acumen.

[bibr42-1471301216682880] Merleau-Ponty M. (1962) Phenomenology of perception, London: Routledge and Kegan Paul.

[bibr43-1471301216682880] Merleau-Ponty M. (1968) The visible and the invisible: Followed by working notes, Evanston, IL: Northwestern University Press.

[bibr44-1471301216682880] Murray S. Kendall M. Boyd K. Sheikh A. (2005) Illness trajectories and palliative care. Clinical Review. BMJ 330: 1007–1111. 15860828 10.1136/bmj.330.7498.1007 PMC557152

[bibr45-1471301216682880] Murtagh, F. E. M., Bauswein, C., Petkova, H., Sleeman, K. E., Dodd, R. H., Gysels, M., … Higginson, I. J. (2012). Understanding place of death for patients with non-malignant conditions: A systematic literature review NIHR. Retrieved from www.netscc.ac.uk/hsdr/files/project/SDO_FR_08-1813-257_V01.pdf.

[bibr46-1471301216682880] Nolan, M., Brown, J., Davies, S., Nolan, J., & Keady, J. (2006). *The SENSES framework: Improving care for older people through a relationship-centred approach*. University of Sheffield. ISBN 1-902411-44-7.

[bibr47-1471301216682880] Nolan M. Davies S. Brown J. Keady J. Nolan J. (2004) Beyond ‘person-centred’ care: A new vision for gerontological nursing. International Journal of Older People Nursing 13(3a): 45–53. 10.1111/j.1365-2702.2004.00926.x 15028039

[bibr48-1471301216682880] Pillow W. (2003) Confession, catharsis, or cure? Rethinking the uses of reflexivity as methodological power in qualitative research. International Journal of Qualitative Studies in Education 16(2): 175–196.

[bibr49-1471301216682880] Robinson C. A. Reid R. C. Cooke H. A. (2010) A home away from home: The meaning of home according to families of residents with dementia. Dementia 9(4): 490–508.

[bibr50-1471301216682880] Ryan T. Nolan M. Reid D. Enderby P. (2008) Using the senses framework to achieve relationship-centred dementia care services: A case example. Dementia 7: 71–93.

[bibr51-1471301216682880] Scottish Government. (2010). Promoting Excellence: A framework for all health and social services staff working with people with dementia, their families and carers. Retrieved from http://www.gov.scot/Publications/2011/05/31085332/0.

[bibr52-1471301216682880] St Christopher’s Hospice Reflective Debriefing. (2013). Adapted from Gibbs’ Model of reflection (1988) & Pearson & Smith experienced-based learning model (1985). Retrieved from www.stchristophers.org.uk/wp-content/uploads/2015/10/practice-development-Reflective-debriefing-tool-updated-Feb-14-pg-1-2.pdf.

[bibr53-1471301216682880] Sudnow D. (1967) Passing on: The social organisation of dying, Englewood Cliff, NJ: Prentice-Hall.

[bibr54-1471301216682880] Tedlock B. (2000) Ethnography and ethnographic representation. In: Denzin N. K. Lincoln Y. (eds) Handbook of qualitative research: Theories and issues,, 2nd ed Thousand Oaks, CA: Sage.

[bibr55-1471301216682880] Thune-Boyle I. C. V. Sampson E. L. Jones L. King M. Lee D. R. Blanchard M. R. (2010) Challenges to improving end of life care of people with advanced dementia in the UK. Dementia 9(2): 259–284.

[bibr56-1471301216682880] Tresolini C. (1994) Health professions, education and relationship-centred care: A report of the Pew Fetzer task force on advancing psychological education, San Francisco, CA: Pew Health Professions Commission.

[bibr57-1471301216682880] Tronto J. C. (1993) Moral boundaries: A political argument for an ethic of care, New York: Routledge.

[bibr58-1471301216682880] Twigg J. (2000) Carework as a form of bodywork. Ageing and Society 20(4): 389–411.

[bibr59-1471301216682880] Twigg J. Wolkowitz C. Cohen R. L. Nettleton S. (2011) Conceptualising bodywork in health and social care. Sociology of Health and Illness 33(2): 171–188. 21226736 10.1111/j.1467-9566.2010.01323.x

[bibr60-1471301216682880] Twigg J. Buse C. E. (2013) Dress, dementia and the embodiment of identity. Dementia 12(3): 326–336. 24336855 10.1177/1471301213476504

[bibr61-1471301216682880] van der Steen J. Radbruch L. Hertogh C. M. P. M. de Boer M. E. Hughes J. C. Larkin P. F. Volicier L. (2013) White paper defining optimal palliative care in older people with dementia: A Delphi study and recommendations from the European Association for Palliative Care. Palliative Medicine 28(3): 197–209. 23828874 10.1177/0269216313493685

[bibr62-1471301216682880] Watson, J. (2015). *Caring with integrity: Developing the conceptual underpinning of relationship-centred palliative dementia care in care homes.* Unpublished PhD Thesis, University of Edinburgh, UK.

[bibr63-1471301216682880] Weiner J. S. (2006) Emotional Barriers to discussing advance directives: Practical training solutions. In: Katz R. S. Johnson T. G. (eds) When professionals weep, London: Routledge, pp. 237–254.

[bibr64-1471301216682880] Whitaker A. (2010) The body as existential midpoint – The ageing and dying body in nursing home residents. Journal of Aging Studies 24: 96–104.

[bibr65-1471301216682880] Zeiler K. (2013) A philosophical defence of the idea that we can hold each other in personhood: Intercorporeal personhood in dementia care. Medical Health Care and Philosophy 17: 131–141. 10.1007/s11019-013-9515-z 24065459

